# Planar Mechanical Metamaterials with Embedded Permanent Magnets

**DOI:** 10.3390/ma13061313

**Published:** 2020-03-13

**Authors:** Viacheslav Slesarenko

**Affiliations:** 1 Freiburg Institute for Advanced Studies, 79104 Freiburg im Breisgau, Germany; sl.slesarenko@gmail.com or viacheslav.slesarenko@frias.uni-freiburg.de; 2 Cluster of Excellence *liv***Mat**S @ FIT—Freiburg Center for Interactive Materials and Bioinspired Technologies, University of Freiburg, 79110 Freiburg im Breisgau, Germany

**Keywords:** metamaterials, mechanical metamaterials, permanent magnets, 3D printing, multistability, bistability, magneto-mechanical metamaterials, architected materials

## Abstract

The design space of mechanical metamaterials can be drastically enriched by the employment of non-mechanical interactions between unit cells. Here, the mechanical behavior of planar metamaterials consisting of rotating squares is controlled through the periodic embedment of modified elementary cells with attractive and repulsive configurations of the magnets. The proposed design of mechanical metamaterials produced by three-dimensional printing enables the efficient and quick reprogramming of their mechanical properties through the insertion of the magnets into various locations within the metamaterial. Experimental and numerical studies reveal that under equibiaxial compression various mechanical characteristics, such as buckling strain and post-buckling stiffness, can be finely tuned through the rational placement of the magnets. Moreover, this strategy is shown to be efficient in introducing bistability into the metamaterial with an initially single equilibrium state.

## 1. Introduction

Mechanical metamaterials possess unique properties and demonstrate unconventional behavior owing to their intricate internal architecture [1]. In many cases, the mechanical properties of the constituents do not play a significant role in defining the overall performance of the metamaterial. Extreme strength can be achieved even in metamaterials made from relatively weak base components due to the rational design of their interior [2]. It resembles the design principles of natural materials and composites that demonstrate extremely high performance despite relatively weak constituents [3,4,5,6]. At the same time, natural materials, as well as some classical mechanical metamaterials, were initially treated as *structural*; therefore, their performance was usually evaluated based on classical mechanical criteria such as strength, stiffness, or toughness [2,7,8,9,10]. However, similar design principles can be employed for the creation of *functional* mechanical metamaterials that are responsive [11], programmable [12], adaptive [13], and smart [14]. 

Various mechanical phenomena have been harnessed to design mechanical metamaterials with intricate functionalities. For instance, it was shown that mechanical buckling could be exploited to amplify the applied deformation and trigger involved reconfiguration of the internal architecture [13,15,16,17,18]. The capacity to program the deterministic deformation sequence through the embedment of the viscoelastic constituents in mechanical metamaterials has been demonstrated both theoretically and experimentally [19]. A similar idea of combining buckling and viscoelasticity inspired the novel design of programmable mechanical metamaterials with memory [20]. Rational selection of the internal architecture enables the development of materials that can convert simple compression into a twisting motion [21,22]. Another emerging design strategy explores the capacity of the internal architecture to transfer local deformation through the metamaterial. In this case of so-called action-at-a-distance metamaterials, the internal architecture may define the involved geometrical changes observed on the surface of the metamaterial in response to applied mechanical stimuli [23,24,25,26].

Nevertheless, the above-mentioned mechanical metamaterials, despite very involved behavior, have one fundamental weakness—their mechanical properties usually cannot be altered after fabrication. To overcome this restriction, the employment of stimuli-responsive constituents has been proposed to provide finer control over the performance of the metamaterials. Indeed, the synergetic combination of a base material capable of reacting to external stimuli by changing its properties or shape, and the rational architecture of the metamaterial to transform these changes into the global deformation/reconfiguration, provides a powerful means to design new adaptive metamaterials [27,28]. For example, the employment of shape memory polymers enables control over the material’s performance by changing the temperature [29]. Moreover, current advances in additive manufacturing techniques promise very quick and simple production and programming of such metamaterials [28]. Another type of stimuli-responsive metamaterials utilizes the ability of the base materials to swell, causing a reconfiguration of the internal architecture, thereby altering the effective properties [30]. At the same time, these two actuation principles (temperature and swelling) are characterized by very high inertia, and therefore it is almost impossible to achieve a high frequency of the actuation.

An alternative external field/stimuli that can be employed to trigger deformation within a metamaterial is the electromagnetic field [11]. Embedding dielectric or piezoelectric elements and magnetorheological elastomers seems to be a feasible strategy to enable control over the performance of these mechanical metamaterials. Application of an external magnetic or electric field can almost immediately trigger the deformation and reconfiguration of the internal architecture, which directly affects the properties and overall performance of the metamaterial [31,32]. However, the current generation of dielectric elastomers/magnetorheological elastomers demonstrates a rather weak coupling between the electromagnetic field and the mechanical deformation [33,34]. Even for the above-mentioned magnetoactive metamaterial [31], a magnetic field of 0.24 T is required to trigger buckling in samples with dimensions not exceeding a couple of centimeters. Such field strength is simply unachievable on a larger scale, making this approach unfeasible for practical applications outside the laboratory. However, there is no doubt that additional magnetic interaction provides powerful means to enhance the functionality of mechanical metamaterials.

Fortunately, there is another strategy to harness the magnetic interaction by employing permanent magnets embedded into the architecture of the metamaterial. This approach was shown to be useful for the origami folding structures, where the employment of permanent magnets enabled the locking of certain states [35]. Other metamaterials employ the interaction between permanent magnets for elastic energy harvesting [36,37]. From a mechanical standpoint, permanent magnets provide a convenient and straightforward way to adjust the stiffness of the lattice structure. Moreover, the insertion of permanent magnets into the structure of a metamaterial can enhance the impact resistance [38]. In practice, mechanical metamaterials consisting of re-entrant unit cells with embedded magnets are able to exhibit negative stiffness and auxetic behavior simultaneously [39]. Moreover, the incremental stiffness of the system can be adjusted by changing the orientation of the magnets [40].

Even in a relatively simple system consisting of magnetic particles connected by elastic springs, advanced phenomena (e.g., multistability) have been observed [41]. More sophisticated systems can be designed by integrating the magnets into the auxetic metamaterials [14,42]. The classical auxetic metamaterial with Poisson’s ratio of −1, which supports the embedment of permanent magnets, consists of rotating squares [43,44] connected by soft hinges. The mechanical behavior of similar metamaterials with rotating units was thoughtfully investigated in multiple publications (e.g., [45,46,47]) in which analytical models that take into account the deformation of the hinges and units were presented and discussed. The embedment of magnetic particles into metamaterials requires introducing additional components into the models to account for a magnetic interaction between elements. For example, the bead-spring model, which takes into account the mechanical force between beads, the Lennard-Jones interaction, and the magnetic dipole–dipole interaction, was shown to be capable of describing the behavior of systems with positive and negative Poisson’s ratios on the nanoscale [48,49]. On a larger scale, similar systems with permanent magnets embedded into the interior of auxetic metamaterials with rotating units were proposed and discussed in [14]. In particular, it was shown that auxeticity and stiffness of mechanical metamaterials with permanent magnets could be tuned by rearranging the magnets and adjusting the external magnetic field. The proposed concepts [14] provide a solid foundation for further theoretical and experimental studies of magneto-mechanical metamaterials.

The present work focuses on mechanical metamaterials with rotating squares in which the behavior is programmed through the modification of the energy landscape by periodic embedment of permanent magnets in various configurations. Here, attention is devoted to the macroscopic properties of the metamaterials (e.g., stiffness and stability), rather than to local structural changes (e.g., rotation angles). The designed planar metamaterials are manufactured using three-dimensional printing and subjected to biaxial compression to obtain continuous force–displacement curves. The selected design enables efficient reprogramming of the mechanical properties by inserting the magnets into specific locations. Numerical simulations support the experimental study and provide the necessary explanation of the observed behavior.

## 2. Materials and Methods

**Figure 1:**
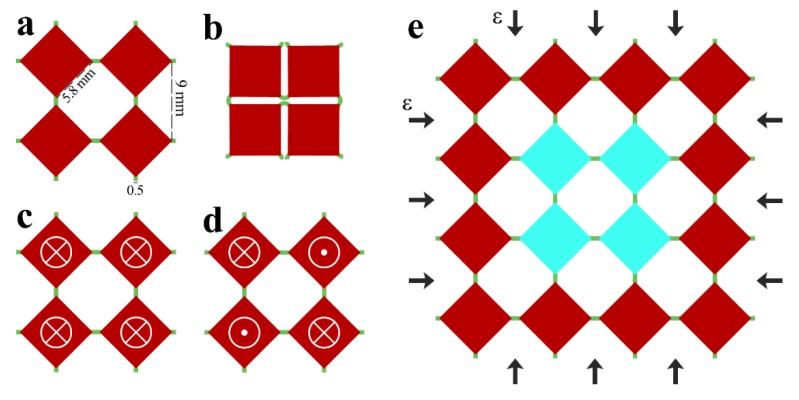
Design of studied mechanical metamaterial. (**a**) elementary cell in the open state; (**b**) elementary cell in the closed state; (**c**) modified (repulsive) elementary cell with co-directional magnets; (**d**) modified (attractive) elementary cell with counter-directional magnets; and (**e**) unit cell with embedded modified cell (shown by blue color) under biaxial compression.

A classical design consisting of rotating squares connected by soft hinges/connectors was adopted in the current study [14,43,44,50]. Figure 1a shows an elementary cell of the metamaterial that consists of four rotating squares. A continuous equibiaxial compression leads to the rotation of stiff squares until the closed state is reached (Figure 1b). Since the goal of this research was to harness magnetic interaction as a tool to program the performance of the metamaterial, some elementary cells were modified through the embedment of permanent magnets at the centers of rotating squares. Three configurations of the elementary cell were considered in this study. Neutral cell did not contain any embedded magnets (Figure 1a), repulsive cell contained four magnets with co-directional out-of-plane magnetic moments (Figure 1c), and attractive cell contained four magnets with counter-directional out-of-plane magnetic moments (Figure 1d). Among various auxetic models [51], this specific design was selected mainly due to the fabrication method and the experimental limitations. 

Numerical simulations of magneto-mechanical metamaterial were performed using FE software COMSOL 5.4a. The plane strain formulation was employed, and out-of-plane thickness of 5 mm was selected to match the dimensions of experimentally tested specimens. The mechanical behavior of the metamaterial with the periodic arrangement of modified elementary cells, which is characterized by the repeated unit cell shown in Figure 1e, was studied. Only four central squares (shown by blue color) were modified by permanent magnets. Selected 4 × 4 unit cell was subjected to equibiaxial compression through the application of the displacement ε on the boundaries (Figure 1e). The corresponding reaction force f was measured through the integration over the boundary of the unit cell. To take into account the periodicity, the equibiaxial compression was implemented using pointwise boundary conditions on the edges of the unit cells as follows:

(1) $$\{\begin{array}{c} \begin{array}{c} u{|}_{left}-u{|}_{right}=\varepsilon a\\ v{|}_{left}-v{|}_{right}=0\;\\ u{|}_{bottom}-u{|}_{top}=0 \end{array}\\ \;v_{|bottom}-v{|}_{top}=\varepsilon a \end{array}\;,$$

where u and v are horizontal and vertical displacements, respectively, ε—applied compressive strain, and a—width and height of the unit cell. 

Rotating squares were treated as rigid non-deformable bodies, while soft connecting hinges were considered as neo-Hookean solids with shear modulus μ=1.1 MPa and Poisson’s ratio of ν=0.46. While FE analysis is capable to take into account the magnetic interaction between permanent magnets, the out-of-plane orientation of the magnetic moments requires 3D formulation and extra meshing of empty space around the metamaterial. An assumption of the dipole–dipole interaction between embedded magnets helps to keep 2D formulation, avoid unnecessary meshing and decrease computation time. Therefore, the magnetic interaction between embedded magnets within one elementary cell was treated as the dipole–dipole interaction [52]. For two identical magnets with out-of-plane orientations of the magnetic moment, the force exerted on the second magnet by the first can be calculated as

(2) $$F_{2}=s_{1}s_{2}\frac{3\mu_{0}m^{2}}{4\pi {\left|r_{12}\right|}^{5}}r_{12},$$

where $\mu_{0}=4\pi \times 10^{-7}$ H/m is the permeability of the vacuum, $r_{12}$ is the vector connecting centers of the first and second magnets, m is the value of the magnetic moment, and $s_{1},\;s_{2}=\pm 1$ define the orientation of the magnetic moment. For convenience $s_{i}=+1$ if the magnetic moment is codirectional with *z*-axis and $s_{i}=-1$ otherwise. According to this definition, two magnets with codirectional out-of-plane magnetic moments experience repulsive forces, while counter-directional orientation corresponds to the attractive configuration. The resulted force exerted on each magnet in modified elementary cell can be obtained as the sum of all pairwise interactions, and it is equal to

(3) $$F_{j}=\frac{3\mu_{0}m^{2}}{4\pi }\underset{i}{\sum }\frac{s_{i}s_{j}r_{ij}}{{\left|r_{ij}\right|}^{5}}\;\mathrm{for}\;i\neq j.$$

Therefore, the magnetic energy of the elementary cell is equal to

(4) $$U=\frac{\mu_{0}m^{2}}{4\pi }\underset{i,j}{\sum }\frac{s_{i}s_{j}}{{\left|r_{ij}\right|}^{3}}\;\mathrm{for}\;i\neq j,$$

where the sum is taken for all pairwise combinations of magnets (for each pair only once). Due to the large distance between modified elementary cells in considered periodic metamaterial, the magnetic interaction between magnets from different cells was neglected. The magnetic forces (Equation (3)) and the magnetic energy (Equation (4)) were manually integrated into COMSOL. 

For the experimental study, mechanical metamaterials were produced with the help of multi-material 3D printer Objet Connex 260 (Stratasys, Eden Prairie, MN, USA). Rotating squares were printed by rigid polymeric VeroWhite (Stratasys, Eden Prairie, MN, USA) material with Young’s modulus around 2 GPa, while soft hinges were produced using soft digital material. A slight modification of the outer boundaries of rotating squares was made in order to ensure the contact between neighbor squares after 45° rotation and to accommodate the deformation of soft hinges without mutual contact. The interior of rotating squares was left hollow to facilitate quick insertion/removal of the magnets after printing. The cubic 5 mm × 5 mm × 5 mm Nd-magnets of N42 grade were used to modify 3D printed metamaterial. The samples were subjected to equibiaxial compression in transparent fixtures preventing out-of-plane deformation, which was attached to the universal testing machine Shimadzu EZ-LX (Shimadzu Corporation, Kyoto, Japan). The tests were performed at a relatively low rate of 10 mm/min in order to mitigate the contribution of the viscoelasticity [53]. A CCD camera was used to capture the deformation sequence. The displacement $D_{b}$ and the force $F_{b}$ acting on the boundaries were calculated as $D_{d}=\frac{D_{m}}{\sqrt{2}}$ and $F_{d}$ = $\frac{F_{m}}{\sqrt{2}}$, where $D_{m}$ and $F_{m}$ are the displacement and the force measured by the testing machine. 

## 3. Results and Discussion

### 3.1. Validation of the Dipole–Dipole Interaction 

**Figure 2:**
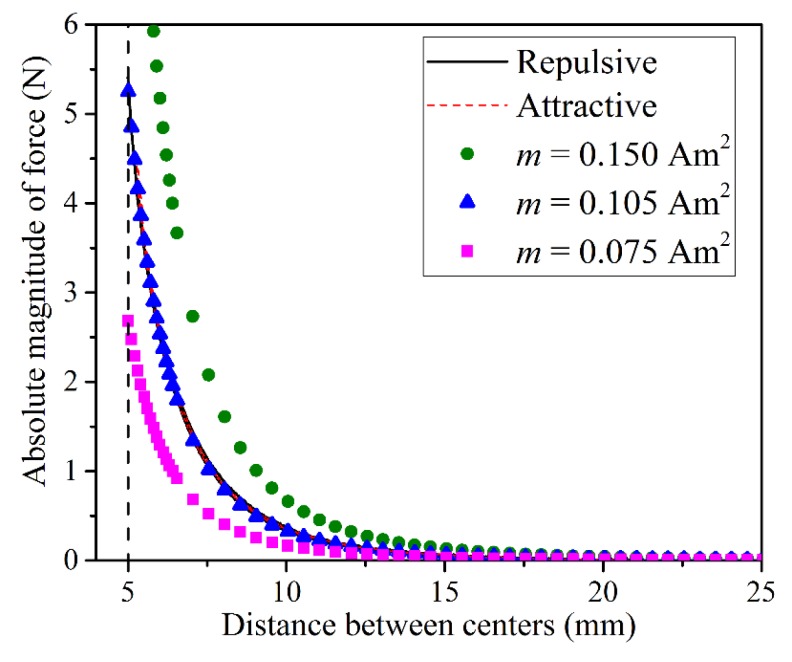
Dependencies of the force between two identical magnets with out-of-plane magnetic moments. Solid lines correspond to experimental measurements, solid symbols represent calculations according to Equation (2) for several values of the magnetic moment.

Auxiliary experimental measurements were performed to explore the accuracy of used dipole–dipole simplification for describing the interaction between cubic magnets with out-of-plane magnetic moments. Two magnets were fixed in 3D printed fixtures attached to the opposite jigs of the universal testing machine. The magnets mounted precisely above each other were slowly moved towards each other, while the displacement of the jigs and the exerting force were measured by the testing machine. At least five different magnets in two configurations (codirectional/repulsive and counter-directional/attractive) were tested. Figure 2 shows the obtained force-distance curves for two tests. As expected, an increase in the distance between magnets led to a drastic decrease in the interaction force both for repulsive and attractive configurations. Solid symbols in Figure 2 correspond to the force-distance relation calculated according to Equation (2) for selected values of the magnetic moment m. As one can see, the estimation obtained for m = $0.105\;{\mathrm{Am}}^{2}$ provided the best fit of the force-distance curves. Note, that this value was lower than the value that could be estimated based on the dimensions and the grade of the magnets as m = $B_{r}V/\mu_{o}\approx 0.127\;{\mathrm{Am}}^{2}$, where V is the volume of the magnet and $B_{r}$ is the remanent magnetization in T. This discrepancy is probably caused by an inaccuracy of the dipole–dipole model for considered configuration, however, a decrease in m value from estimated $m=0.127\;{\mathrm{Am}}^{2}$ to $m=0.105\;{\mathrm{Am}}^{2}$ enables a very good fit of experimental curves, therefore the dipole–dipole interaction model with $m=0.105\;{\mathrm{Am}}^{2}$ was adopted in the following numerical section. 

### 3.2. Numerical Results for Periodic Metamaterials

**Figure 3:**
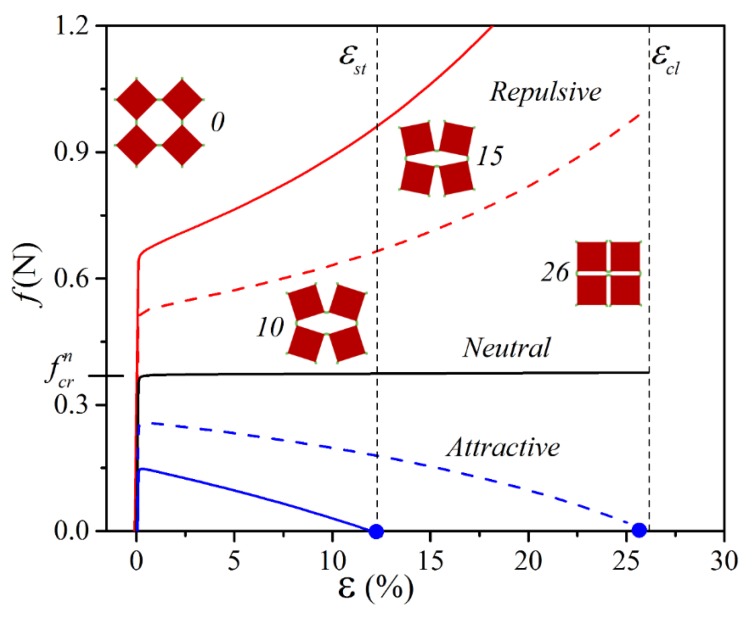
Numerically obtained force–strain curves for the metamaterial with unit cell (Figure 1e) modified by the embedment of the magnets in repulsive and attractive configurations. Solid lines correspond to the results for the magnets with $m=0.105{\;\mathrm{Am}}^{2}$, dashed lines for the magnets with $m=0.075{\;\mathrm{Am}}^{2}$. Blue circles on the horizontal axis denote the strain $\varepsilon_{st}$ corresponding to the snap-through phenomena. The numbers near the snapshots represent applied strain ε.

Figure 3 shows the force–strain curves obtained numerically for periodic metamaterials with various configurations of elementary cells (neutral, repulsive, and attractive). Additional dashed curves are shown for the metamaterial with weaker embedded magnets ($m=0.075{\;\mathrm{Am}}^{2})$. The metamaterial in neutral configuration underwent buckling upon reaching critical force $f_{cr}^{n}$ that was accompanied by rotation of rigid squares in alternating directions. As a result, an initial compressive state in soft hinges was replaced by bending during post-buckling deformation, causing the drastic drop in the slope of the force–strain curve, as shown in Figure 3. The rotation continued with an increase in applied strain ε until all squares rotated by 45° degrees, and the metamaterial completed its transition to the closed state (Figure 1b), when $\varepsilon =\varepsilon_{cl}$. Mechanical metamaterials with embedded magnets demonstrated a similar bucking behavior and underwent softening after buckling at the early stage of the deformation. At the same time, the critical force required to trigger the buckling depended on the configuration and strength of the magnets. Indeed, embedment of the magnets in the repulsive configuration led to an increase in the critical force, while an opposite effect was observed for the system with attractive elementary cells. Moreover, it is clear that the deviation from $f_{cr}^{n}$ increased with an increase in the value of the magnetic moment. 

Figure 3 reveals that the slope of the force–strain curve in the post-buckling regime was also highly affected by the configuration of embedded magnets. Through the comparison with neutral configuration, it is easy to notice that the contribution of the magnetic interaction in repulsive configuration led to a stiffer response of the metamaterial. Surprisingly, attractive configuration resulted in the negative stiffness after buckling, and therefore a lower force needed to be applied in order to continue equibiaxial compression. Numerical simulations revealed that for repulsive configuration of magnets and large enough m, the decaying force could reach zero at some value of strain $\varepsilon_{st}$ prior to the achievement of the strain $\varepsilon_{cl}$. In this case, a rapid snap-through transition to the closed state occurred, and the metamaterial remained in the closed state even after the removal of external load.

**Figure 4:**
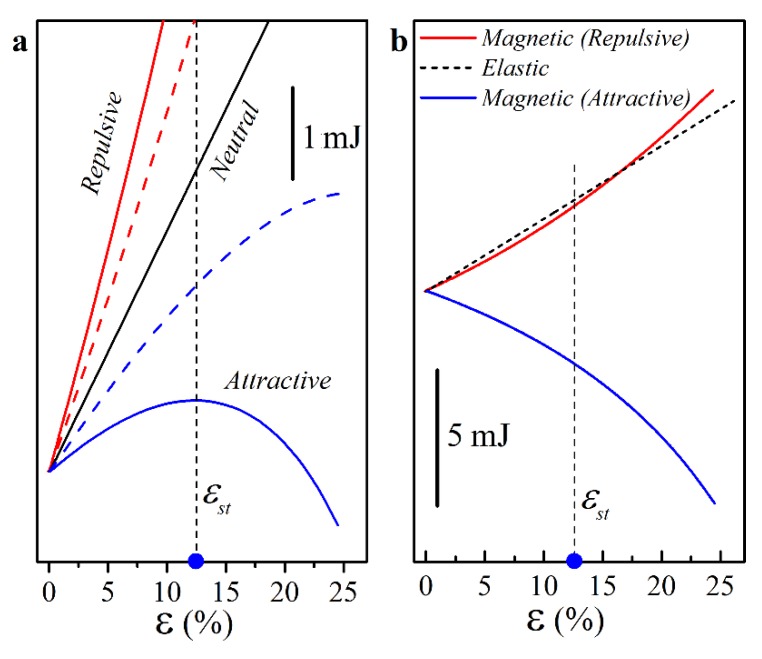
Numerically obtained dependencies of the energy of the unit cell on the applied strain ε. (**a**) Total energy vs. ε for various configurations of magnets. Solid lines correspond to the magnets with $m=0.105{\;\mathrm{Am}}^{2}$, dashed lines to the magnets with $m=0.075{\;\mathrm{Am}}^{2}$. (**b**) Magnetic energy for repulsive configuration (red), attractive configuration (blue), and elastic energy for neutral configuration (dashed). Note that all energy curves are shifted vertically to have a (0, 0) origin.

A closer examination of the energy of the unit cell provided a clear explanation of the observed behavior. The total energy of the system $E_{total}$ can be evaluated as the sum of magnetic energy $\left(E_{mag}\right)$ and elastic energy stored in soft deformable hinges $\left(E_{el}\right)$. Elastic energy $E_{el}$ was calculated by integrating the strain energy density function over soft hinges. Figure 4a shows the dependencies of the total energy of the system on applied strain ε, while Figure 4b shows both components of the total energy for repulsive and attractive configurations with $m=0.105{\;\mathrm{Am}}^{2}$. For neutral configuration, the total energy increased monotonically due to an increase in $E_{el}$ during deformation. The addition of repulsive configuration led to an increase in the total energy, since $E_{mag}$, calculated according to Equation (4), had a positive sign. During the deformation, $E_{mag}$ increased monotonically, contributing to the growth of $E_{tot}$. Therefore, $E_{tot}$ as a function of the applied deformation had only one minimum corresponding to the open state of the metamaterial. An opposite observation could be made for the attractive configuration. The magnetic energy $E_{mag}$ had a negative sign, and the biaxial compression led to a decrease in $E_{mag}$ due to the decrease in the distance between the magnets in elementary cell, as shown in Figure 4b. As a result, the total energy of the system $E_{total}=E_{mag}+E_{el}$ became concave, and for large enough m, had a maximum for the strain $\varepsilon_{st}<\varepsilon_{cl}$, marked by the blue circle in Figure 4. Therefore, under these conditions, $E_{total}\left(\varepsilon \right)$ had two local minima, separated by a potential barrier, that corresponded to the open and closed states of the metamaterial. As soon as the applied strain ε reached the critical value $\varepsilon_{st}$ during equibiaxial compression, the metamaterial snapped into the second equilibrium state. Therefore, the rational embedment of interacting permanent magnets can be exploited to modify the energy landscape of the metamaterials and to create systems with multiple stable equilibrium states.

### 3.3. Experimental Results

**Figure 5:**
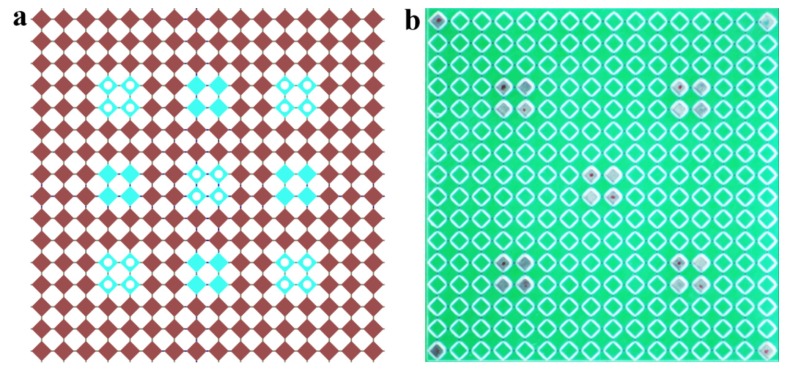
Arrangements of the magnets in 16 × 16 metamaterial used for experiments and simulations. (**a**) Blue squares mark the positions of the magnets for the arrangement I, white circles mark the positions for the arrangement II. (**b**) Example of 3D printed metamaterial with embedded magnets (arrangement II). Four magnets in the corners are used for the mounting in the fixtures and do not affect the behavior of the metamaterial during tests.

**Figure 6:**
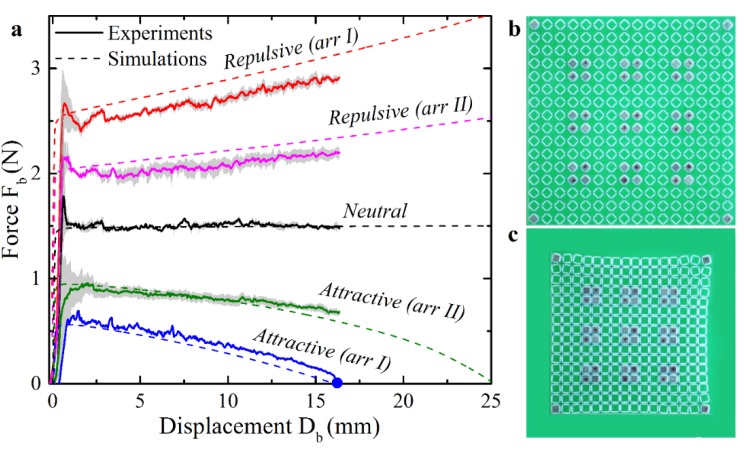
(**a**) Force–displacement curves obtained experimentally (solid lines) and numerically (dashed lines) for finite 16 × 16 metamaterials with arrangements I and II. Solid lines show the means for three consecutive loadings of the metamaterial, while gray areas correspond to the standard deviation. (**b**,**c**) Two stable states of the metamaterial (arrangement I), observed experimentally. The snap-through occurs at the displacement marked by the blue circle (Figure 6a).

For the experimental study, the metamaterials consisting of 16 × 16 rotating squares with two various arrangements of the magnets were considered. Arrangement I closely resembled the design used in the numerical study with the positions of the magnets shown in Figure 5a by blue squares. Arrangement II had magnets only at the diagonal locations, as shown by white circles in Figure 5a. Figure 6a shows experimentally obtained force–displacement curves (two for each arrangement with repulsive and attractive configurations, plus neutral metamaterial). Note, that the metamaterials were compressed only up to 17 mm (ε=0.12) to prevent possible failure of the hinges due to high local strains and relatively weak 3D-printable material. Dashed lines represent the additional numerical simulations performed on the finite 16 × 16 metamaterials with arrangements I and II. As one can see, experimental curves very closely match the numerical predictions with respect to the buckling force and the slope of the curves in the post-buckling regime. Similar to the numerical results, the significant softening and even negative effective stiffness were observed experimentally. Figure 6a also revealed that the increase/decrease in the critical buckling force for repulsive/attractive configurations relative to the neutral configuration was observed irrespectively of the magnet arrangement. At the same time, the metamaterial with the arrangement II demonstrated a lower deviation of the buckling force in comparison with the arrangement I due to a lower number of the modified elementary cells. The contribution of the magnetic energy towards the total energy of the system is directly proportional to the density of modified elementary cells in the metamaterial. Therefore, the employment of stronger magnets and the embedment of a larger number of magnets are two feasible strategies in amplifying the effect of the magnetic interaction on the overall performance of mechanical metamaterial. 

During the equibiaxial loading of the specimens with attractive magnets, the force–displacement curves demonstrated the negative slope in the post-buckling regime. For arrangement II, the force remained positive during biaxial compression, and the removal of external load led to the restoration of the open state (Video S1 in Supporting Materials). On the contrary, the experimental study confirmed that for arrangement I (Figure 6b), when the measured force dropped to almost zero, the snap-through transition to the closed state was observed (Figure 6c and Video S2 in Supporting Materials). The sample remained in the closed state even after the removal from the fixtures proving that this closed state is another stable equilibrium. It is clear that the value of $\varepsilon_{st}$ depends on the arrangement of the magnets and their strength. Therefore, by adjusting the position or the density of the modified elementary cells in the metamaterials, it seems possible not only to create the second stable state, but also to adjust the critical strain $\varepsilon_{st}$ at which the snap-through transition between states occurs.

## 4. Conclusions

Here, the strategy to program and control the mechanical behavior of planar mechanical metamaterials through the embedment of the interacting permanent magnets was studied. On the example of the metamaterial with rotating squares, the advantages of this strategy were shown both experimentally and numerically. The planar metamaterial produced by three-dimensional printing enables efficient and fast adjustment of the mechanical properties by rearranging the magnetic cells. By utilizing this design, the control over the buckling strain and the stiffness of the system in the post-buckling regime was demonstrated experimentally. It was also experimentally confirmed that the rational placement of the attractive magnets could be harnessed to introduce multistability into the metamaterial with an initially single equilibrium state. By generalizing the observations, it can be concluded that the underlying strategy enabling the enhanced control over the behavior of mechanical metamaterials is based on the thoughtful modification of the energy landscape by employing intricate interplay between the elastic and magnetic energies. More sophisticated auxetic models enabling larger changes in the internal structure (e.g., [54]) can be considered as promising candidates to host permanent magnets, but the feasibility of their fabrication should be taken into account. With the rapid development of additive manufacturing techniques, similar design principles can be potentially applied for three-dimensional auxetic systems to design advanced mechanical metamaterials with programmable behavior.

## Supplementary Materials

The following are available online at https://www.mdpi.com/1996-1944/13/6/1313/s1. Video 1: Equibiaxial compression of mechanical metamaterial with attractive magnets in the arrangement II, Video 2: The snap-through phenomenon during equibiaxial compression of mechanical metamaterial with attractive magnets in the arrangement I.
