# Supplementary material for: Planar Mechanical Metamaterials with Embedded Permanent Magnets
Source: Materials (Basel). 2020 Mar 13;13(6):1313. doi: 10.3390/ma13061313 (PMC7143886; doi:10.3390/ma13061313)
Supplement: Supplementary file 1 [file materials-13-01313-s001.zip › supplementary_materials.pdf]

*materials*

MDPI

# Supplementary materials for “3D printed Mechanical Metamaterials with Embedded Permanent Magnets”

**Viacheslav Slesarenko** [1,2,*]

[1] Freiburg Institute for Advanced Studies, Freiburg im Breisgau, Germany 79104
[2] Cluster of Excellence *liv*MatS @ FIT – Freiburg Center for Interactive Materials and Bioinspired Technologies, University of Freiburg, Freiburg im Breisgau, Germany 79110
* Correspondence: sl.slesarenko@gmail.com or viacheslav.slesarenko@frias.uni-freiburg.de

These two videos demonstrate the equibiaxial compression of the 3D printed mechanical metamaterial with embedded magnets in the attractive configuration. Video 1 corresponds to the arrangement II from the main manuscript, for which the return to the initial stable state occurs after unloading. Video 2 corresponds to the arrangement I, for which a snap-through phenomenon is observed. Both videos are sped up by x20.
